# The 2021 Chronic Kidney Disease Epidemiology Collaboration Race-Free Estimated Glomerular Filtration Rate Equations in Kidney Disease: Leading the Way in Ending Disparities

**DOI:** 10.1089/heq.2023.0038

**Published:** 2024-01-12

**Authors:** Keyerra Charles, Mary Jane Lewis, Elizabeth Montgomery, Morgan Reid

**Affiliations:** National Kidney Foundation, New York, New York, USA.

**Keywords:** chronic kidney disease, health disparities, 2021 CKD-EPI race-free eFGR equations, health equity

## Abstract

**Purpose:**

In 2020, the National Kidney Foundation (NKF) and the American Society of Nephrology (ASN) convened a Task Force to recommend an evidence-based race-free approach to estimated glomerular filtration rate (eGFR). After the rigorous review of more than 20 approaches, the NKF/ASN Task Force published the final report that recommended the implementation of the Chronic Kidney Disease Epidemiology Collaboration (CKD-EPI 2021) equation for eGFR using creatine and expanded utilization of cystatin C testing. The purpose of this manuscript is to provide a comprehensive overview of the evolution of eGFR equations, and an overview of the Task Force deliberations and recommendations. For over two decades, the equation recommended to calculate eGFR included a race coefficient to adjust for data that suggested that American adults with African ancestry had consistently higher serum creatinine levels.

**Methods:**

We will provide a discussion illustrating why the 2021 CKD EPI equations are the most equitable solution to eGFR. We will also provide an overview of the current implementation status and best practices for the new equations. Lastly, we will discuss how deployment of the new equations is an important step toward eliminating significant disparities in CKD care which disproportionately affect communities of color.

**Results:**

Removing race from the algorithm used to assess kidney function is most equitable. Since race is a social construct, its use in clinical algorithms has facilitated health disparities in Black/African American people, Hispanic/Latino people, and other racial and ethnic minority groups—those who are already disproportionately impacted by diabetes, hypertension, and kidney disease. In turn, these same individuals experience significant inequities in kidney health care including reduced access to nephrology care, home dialysis, and kidney transplant.

**Conclusions:**

Adoption of the race-free 2021 CKD-EPI eGFR equations will have life changing implications for kidney health. It will aid in appropriate referral, identification, diagnosis, treatment, and management of kidney disease and transplantation services/options. The outcomes of widespread implementation of the new equations coupled with system change quality improvement interventions such as the kidney profile will lead to more equitable outcomes and begin to address the crippling disparities in early, appropriate testing for CKD.

*Of all the forms of inequality, injustice in health care is the most shocking and inhumane.—Dr. Martin Luther King, Jr.*

## Introduction

The trajectory of health care outcomes are heavily influenced by both biological and nonbiological factors which shape diagnoses and prognosis of disease. Differences in these factors fuel health care disparities and have historically impacted the variability in disease journeys between ethnic groups. In the case of chronic kidney disease (CKD), many nonbiological factors stemming from historical systemic barriers and racism propel the current status of CKD impact in the United States.^1^ CKD is associated with significant morbidity, cardiovascular mortality, and costs.^2,3^ According to the Centers for Disease Control and Prevention^4^ (CDC), 37 million adults in the United States are estimated to have CKD, and 90% are undiagnosed. CKD is more common in non-Hispanic Black adults (16%) than in non-Hispanic White adults (13%) or non-Hispanic Asian adults (13%), and (14%) of Hispanics adults.^4^

Racial and ethnic minority groups and individuals with low socioeconomic status experience worse kidney health and clinical outcomes, for example, CKD is 3.8 times more prevalent in Black/African American individuals, and they spend almost 2 times longer on waitlists for dialysis and transplant compared to White individuals.^5^ Additionally, Hispanic/Latino individuals are at a 2.1 times higher risk for kidney failure and are 1.3 times less likely to receive a kidney transplant compared to White individuals.^5^ The scale of the population affected and the disparities that exist within the prevalence of CKD further support the need for routine, unbiased estimation of kidney function among those at increased risk (i.e., people with obesity, diabetes, hypertension, over the age of 60, or with a family history of kidney disease).

Included in the ubiquitous Basic Metabolic Profile and Comprehensive Metabolic Profile laboratory test panels, serum creatine with estimated glomerular filtration rate (eGFR_cr_) is one of two tests used to detect and assess kidney disease.^6^ For over 20 years, the equations used to estimate eGFR have employed a race variable in their calculation: the 1999 Modification of Diet in Renal Disease Study eGFR_cr_, the Chronic Kidney Disease Epidemiology Collaboration (CKD-EPI) 2009 eGFR_cr_, and the CKD-EPI 2012 eGFR_cr-cys_equations.^7,8^ The coefficient paints a picture of race-driven medicine that can be perceived by individuals as a form of discrimination.

The overall impact on an individual patient will be relatively small but in general those self-identifying as African American will have slightly lower eGFR and all other racial groups will have slightly higher eGFR when using the new race-free CKD EPI equations versus the older equations with race coefficients. National conversation about racial equity and recognition that race is a social construct and not a biological one has focused attention on the need to remove race in clinical equations.

In response to this call to action, the National Kidney Foundation (NKF) and the American Society of Nephrology (ASN) created the Task Force on Reassessing the Inclusion of Race in Diagnosing Kidney Diseases to examine the issue and provide recommendations. The Task Force systematically evaluated 26 approaches (4 of which were not creatine based) for GFR estimation that did and did not include race, and then by consensus narrowed its focus to 5.^9^

**FIG. 1.**
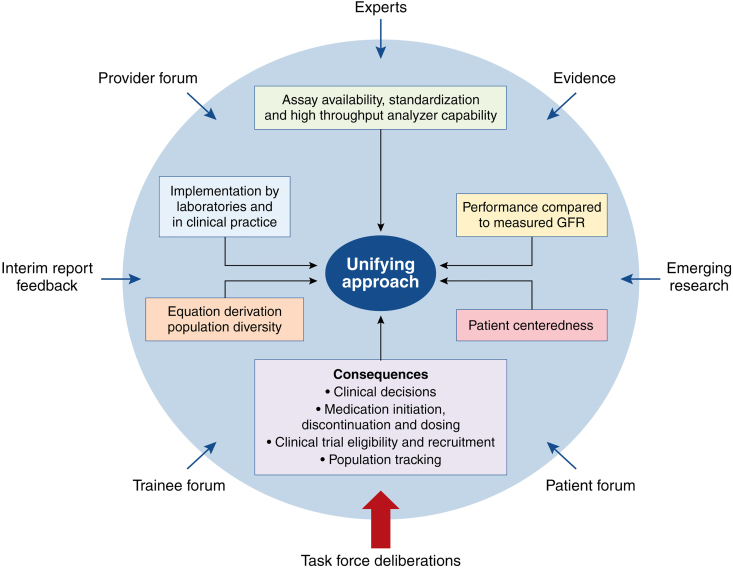
Process and input to create a unifying approach to GFR estimation. This comprehensive strategy used sources (*blue arrows*) to identify and evaluate attributes (*boxes*) of 26 approaches. Each source provided information about multiple topics: equity and disparities; race and racism; GFR measurement, estimation, and equation performance; laboratory standardization; consequences; patient perspectives; and new science. Information was integrated by the Task Force in its considerations of the attributes during deliberations to arrive at a unifying approach. Note: This figure was produced by the NKF and American Society of Nephrology Task Force. Copyright 2021 NKF. NKF, National Kidney Foundation.

Each was assessed holistically and considered six characteristics: (1) availability and standardization; (2) implementation; (3) population diversity in equation development; (4) performance compared with measured GFR; (5) consequences to clinical care, population tracking, and research; and (6) patient centeredness.^9^ To arrive at a unifying approach to eGFR (Fig. 1), the Task Force incorporated information and evidence from many sources to assess strengths and weaknesses for each equation, recognizing the number of Black and non-Black adults affected.^9^ An overview of the Recommendations released on September 23, 2021:

1. Immediate implementation of the CKD-EPI 2021 creatinine equation refit without the race variable in all US laboratories.
2. National efforts to facilitate increased, routine, and timely use of cystatin C, especially to confirm eGFR in adults who are at risk for or have CKD.
3. Research on GFR estimation with new endogenous filtration markers and on interventions to eliminate race and ethnic disparities should be encouraged and funded.^9^

To facilitate implementation of these recommendations by the laboratory community, the NKF engaged its Laboratory Engagement Initiative (LEI) Workgroup, a collaboration of national pathology societies and national and academic laboratories established in 2016 to understand and address factors causing low and incomplete CKD testing and promote best practices in laboratory services related to CKD. In partnership with the members of the LEI, NKF developed the implementation strategies needed to ensure the rapid deployment of the new race-free CKD EPI 2021 equations. Members of the LEI helped engage the leadership of the LOINCs^®^ (Logical Observation Identifiers Names and Codes) Laboratory committee.^10^

LOINCs is a common coding system that laboratories and clinical systems use for identifying health data that are important to population health activities and clinical trials. This outreach and collaboration resulted in the LOINC Laboratory Committee expediting publication of the new LOINC codes for the CKD-EPI 2021 equations by late September 2021 to facilitate the rapid implementation of the new equations. The endorsement from the leaders in the laboratory community reflected the importance of the clinical impact of this change and is evidenced by the speed with which the NKF was able to secure LOINC codes, a process that normally requires a minimum of 6 months was reduced to a few days.

As deploying a new equation in a clinical laboratory can be a complex, time-consuming undertaking, the NKF LEI Workgroup authored practical guidance for clinical laboratories implementing the new equations, which were published in *Clinical Chemistry*^7^ in December 2021. To fill the gap between publication of the CKD-EPI equations and the time required for its institutional implementation, NKF ensured that its online eGFR calculator^11^ and eGFR app^12^ were updated and available for immediate use, in addition to readily available written resources explaining the new equation for clinicians and consumers.

## Methods

Employing an equitable clinical algorithm to assess kidney function is essential in the healthcare field. We reviewed and discussed the NKF/ASN Task force recommendations as well as the summary of findings from the awareness and adoption surveys administered by the College of American Pathology to gain a better understanding of the implementation status among various laboratories. We explore the importance of adopting an equitable equations to assess kidney function. Additionally, we provide programmatic and quality improvement support tools to aid in the adoption of the new equations.

## Results

In the fall of 2022, the NKF surveyed laboratory awareness and adoption of the recommended race-free eGFR equations through collaborations with the Association of Pathology Chairs (APC) and the American Society for Clinical Pathology (ASCP). These survey outcomes paralleled those of the College of American Pathologists (CAP) survey, which was administered in March 2022 and summarized in the *Journal of the American Medical Association* in November 2022.^13^ In 2023, CAP released updated adoption results, reporting that 65.8% of laboratories surveyed had already adopted the 2021 CKD-EPI creatine equation and 7.3% had already adopted the 2021 CKD-EPI creatine-cystatin c equation, citing a significant increase from the previously published 2022 results.^14^

Implementation of the new equations eliminates potential race disparity concerns and offers laboratories the opportunity to standardize eGFR reporting which, in turn, facilitates more consistent practice of patient management across the United States. Particularly, the implementation of the new equations will lead to sooner eligibility for dialysis and transplant compared to for Black/African American patients.^5^

The recommendations serve as a framework to standardize care^15^ and improve equitable practices within the health care system. Health disparities are fueled by generations of racial inequities and a re-evaluation of the inclusion of race in clinical algorithms is an important step toward achieving health equity. Clinical algorithms and tests used to diagnose, treat, and recommend care should no longer continue to risk population health by including a nonbiological component such as race to aid in the prediction of health status. Ultimately, race has no place in the assessment of and testing for kidney disease and should not be a driver for decision-making regarding care.

Adopting the 2021 CKD-EPI creatine equation and expanding utilization of cystatin C testing recommendations nationally are best practices to advance health equity in kidney care, including sooner eligibility of dialysis and transplant for Black/African-American patients. The increase in cystatin C testing will facilitate more frequent use of the 2021 CKD-EPI_cr-cys_ equation. The latter was shown to be more accurate and led to smaller differences between Black patients and non-Black patients than new equations without race with either creatine or cystatin C alone.^8^

Supporting a widespread shift in medical practice requires collective effort and advocacy on every level of influence in health care. A unanimous decision by the Organ Procurement and Transplantation Network board mandating all U.S. Kidney Transplant centers to readjust waitlist times based on implementation of the race-free eGFR equations^16^ for Black kidney patients who were originally assessed using the race-based algorithm confirms a concerted effort to revisit existing practices that have hindered positive health outcomes for different groups of people.

NKF leads coordination of the implementation of the new race-free equation among American laboratories through development and implementation of a tool kit^17^ along with several other initiatives to address disparities in kidney care and the potential challenges (i.e., IT resources, and budget allocation) that mid- and smaller-scale laboratories (including academic hospitals, physician-owned, and hospital health system labs) may face in implementing these recommendations.

The implementation of the new race-free CKD-EPI 2021 equations, while important, is simply the first step to improving CKD care. The adoption of the equations will begin to move the needle on individuals receiving appropriate diagnoses and early management of their CKD. Additionally, there are tools and strategies that can be deployed across health systems to support clinicians and care teams to increase guideline concordant testing and diagnosis, including electronic health record clinical decision support tools or patient registries, standing lab orders, or facilitated clinician practice change sessions (i.e., Project ECHO).^18–21^ If organizations additionally commit to internal assessment of CKD care gaps and utilization of practice change strategies to close care gaps amplified by use of the new equation, we will make sustainable impact on the CKD care.

**FIG. 2.**
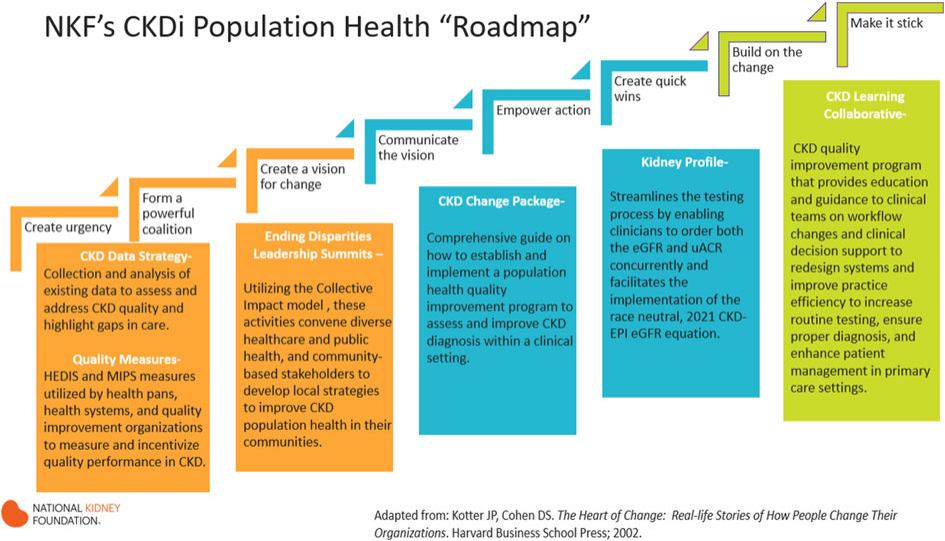
The NKF's CKDi Population Health “roadmap.” Displaying the CKDi programs. CKDi, Chronic Kidney Disease Intercept.

NKF's CKD *Intercept*^22^ (CKDi) offers various program components aimed at assisting institutions who are looking to do more than just swap the equations and actually amplify impact of overarching quality of care. These programs (Fig. 2) aid health care partners and stakeholders in the implementation of the new equations while working to ensure that the new equations and its impact on health disparities are explained.

CKDi includes the LEI Workgroup which focuses on the implementation of the new equations and the Kidney Profile.^23^ The Kidney Profile provides health care practitioners a convenient, seamless method for ordering the two tests eGFR (assesses kidney function) and urine albumin-creatine ratio (uACR) which assesses kidney damage both of which are needed in the identification and risk stratification of CKD. While eGFR is frequently available, only 21% of people at risk for CKD receive uACR testing annually.^24^ As albuminuria is associated with higher rates of CKD^25–29^ progression and can be present before the eGFR declines, administration of both tests is necessary to accurately determine the severity of CKD. NKF engages with national laboratory and pathology organizations, including but not limited to CAP,^30^ ASCP, APC, Clinical Laboratory Improvement Advisory Committee (CLIAC), etc., to advance the implementation of task force recommendations that, in turn, will drive sustainable changes to improve kidney care.

To improve awareness of the scale of underdiagnosis, its impact on the community and the inequities that exist in kidney disease, including the continued utilization of race-based eGFR calculations, the NKF's CKDi is advancing *The Ending Disparities Leadership Summits*.^31^ This convening leverages the collective impact model^32^ to engage senior leadership from across the stakeholder spectrum (health care executives, payers, community-based organizations, employers, and people living with kidney disease) in a conversation about the barriers that exist to improve CKD care and the strategies that the community can employ to address them. Each of these events has led to significant community commitment to advancing change in CKD care and improving health equity, including engagement of local health care institutions in the deployment of the CKD Data Strategy.

NKF's CKD Data Strategy engages institutions (integrated delivery networks, federally qualified health centers, individual practices, etc.) in looking at existing electronic health care data to illuminate gaps in CKD testing, diagnosis, risk stratification, and management. The outcome of this process is an organizational scorecard for CKD and specific recommendations for leveraging the NKF's CKD Change Package tools and resources to address identified gaps in care (such as the implementation of the new equations, recommendations for CKD clinical decision support, update of Electronic Health Record tools (i.e., coded phrases).

## Conclusion

Adoption of the race-free 2021 CKD-EPI eGFR equations will have life-changing implications for kidney health. It will aid in appropriate referral, identification, diagnosis, treatment, and management of kidney disease and transplantation services/options. According to the *Journal of the American Society of Nephrology*,^33^ the new equation, if applied nationally, will allow for the reclassification of 584,000 Black Adults to more advanced stages of CKD, and expand nephrologist referral for 41,800 among other implications specific to kidney donation eligibility.

Furthermore, the race-free 2021 CKD-EPI equations has acceptable performance characteristics and potential consequences that do not disproportionately affect any one group of individuals.^8^ Consequences that the NKF/ASN taskforce considered are CKD screening and detection, nephrology referral, drug dosing, outcome risk assessment among others.^9^ As the nation continues to address racial and health disparities, the race-free CKD-EPI 2021 eGFR equations adoption is on the forefront of establishing sustainable change in health outcomes for adults with kidney disease. The NKF is committed to identifying and dismantling systemic barriers in place that contributes to inequity in kidney health.

The adoption of the new race-free equation is moving its way across the nation and beyond.^34^ However, there is still a lot of work to be done to ensure that all medical institutions, laboratories, and ordering providers see the value of the new equations, implement it and then follow implementation with wide spread use of the kidney profile to address crippling disparities in early, appropriate testing for CKD. The outcome of a nationwide shift to the new equation will lead to more equitable outcomes for those impacted by kidney disease.

The NKF continues to live out the mission while always keeping the 37 million people impacted by kidney disease as its focus. Making strides to address racial and health equity barriers are a part of the important work the NKF's is committed to.

## Abbreviations Used

APC: Association of Pathology Chairs
ASCP: American Society for Clinical Pathology
ASN: American Society of Nephrology
CAP: College of American Pathologists
CDC: Centers for Disease Control and Prevention
CKD-EPI 2021: Chronic Disease Epidemiology Collaboration
CLIAC: Clinical Laboratory Improvement Advisory Committee
eGFR: estimated glomerular filtration rate
EHR: Electronic Health Record
LEI: Laboratory Engagement Initiative
NKF: National Kidney Foundation
uACR: urine albumin-creatine ratio
